# Hyperviscosity Syndrome in Undifferentiated Connective Tissue Disease: A Diagnostic and Therapeutic Challenge

**DOI:** 10.7759/cureus.55399

**Published:** 2024-03-02

**Authors:** Ian Mines, Alaq Al-Abayechi, Supreet Kaur, Zohra Nooruddin

**Affiliations:** 1 Internal Medicine, University of Texas Health Science Center at San Antonio, San Antonio, USA; 2 Hematology and Medical Oncology, University of Texas Health San Antonio MD Anderson Cancer Center, San Antonio, USA

**Keywords:** undifferentiated connective tissue disorder (uctd), epistaxis, rash, plex, hyperviscosity syndrome

## Abstract

Hyperviscosity is an uncommon manifestation of various underlying diseases. Rapid diagnosis and management of the underlying disease is crucial to prevent significant complications, including hypertension, cerebral vascular accidents, pulmonary embolism, bowel ischemia, and ophthalmologic pathologies. Although the acute management of complications arising from hyperviscosity is relatively straightforward, identifying and treating the underlying cause can prove difficult. This case highlights the difficulties of establishing a diagnosis and initiating appropriate management for a patient with hyperviscosity syndrome in a suspected rheumatologic disorder.

## Introduction

Hyperviscosity is a rare clinical manifestation of various disorders and requires a broad differential. The etiology of hyperviscosity falls within three disease categories: primary hematologic, infectious, and rheumatologic [1-4]. The diagnostic requirements for hematologic and infectious processes are relatively concrete and require only one to two positive laboratory and/or pathologic findings [1]. Diagnosing an underlying infection is typically confirmed by culture or polymerase chain reaction (PCR) testing. In hematologic-associated hyperviscosity, typically, a biopsy can confirm the diagnosis. However, diagnosing an underlying rheumatologic disease requires a much higher diagnostic threshold and poses a therapeutic challenge, which often delays diagnosis and treatment [2-3,5-8].

The diagnosis of hyperviscosity syndrome and its etiology are twofold. First, one must identify whether the patient has hyperviscosity syndrome. This includes evaluating for signs and symptoms of hyperviscosity, determining its severity, and treating with plasma exchange or apheresis therapy in the acute setting if needed [1,4]. It is vital to perform an ophthalmologic evaluation in patients with hyperviscosity, as central retinal vein occlusion can occur and lead to permanent blindness. Second, the underlying etiology must be determined to prevent further hyperviscosity episodes. The most common etiologies of hyperviscosity syndrome include multiple myeloma, Waldenstrom macroglobulinemia (WM), acute leukemia, myeloproliferative diseases, Sjogren’s disease, rheumatoid arthritis, systemic lupus erythematosus, human immunodeficiency virus (HIV), cryoglobulinemia, and homozygous sickle cell anemia [1,4].

This case report highlights the extensive diagnostic workup needed to evaluate hyperviscosity syndrome, its associated etiologic disease, and acute management.

## Case presentation

**Figure 1:**
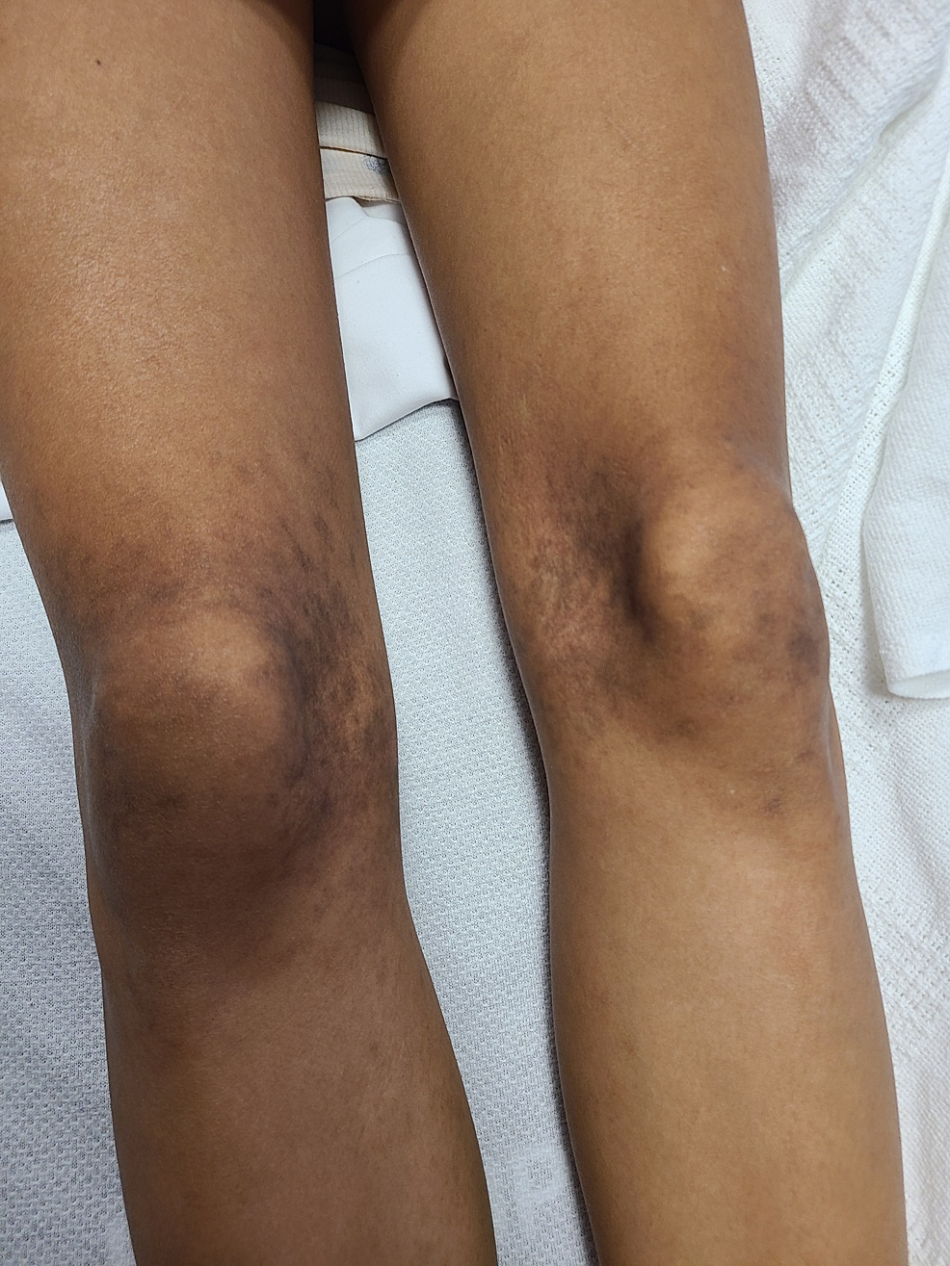
Rash of the medial knees

A 19-year-old Hispanic female with no past medical history, who takes no supplements and without illicit drug use, presented with 15-pound weight loss over the past year, recurrent nose bleeds, bleeding with tooth brushing, lower extremity proximal weakness without muscle pain, and hyperpigmented rash on the posterior aspects of the thighs, medial knees, and dorsum of the foot (Figure 1).

Vital signs were remarkable for a BMI of 16.46. Initial labs were consistent with iron deficiency anemia, mild transaminitis, elevated total protein, low albumin, and elevated creatinine kinase (Table 1). A CT scan of the chest and abdomen was significant for splenomegaly measuring 14 cm and bilateral axillary lymphadenopathy. A peripheral blood smear showed hypochromic microcytic anemia with marked rouleaux formation and elevated serum viscosity. The patient later developed blurry vision, which prompted an ophthalmologic consult and exam that showed bilateral dot and blot hemorrhages with venous tortuosity. The patient then underwent treatment with two doses of plasma exchange therapy to acutely manage her hyperviscosity syndrome. However, the long-term treatment of hyperviscosity relies on determining the underlying etiology and disease process. Therefore, additional hematologic, rheumatologic, and infectious workups were initiated. The differential diagnosis at this time was broad and included chronic lymphocytic leukemia, chronic myelocytic leukemia, acute myelocytic leukemia, lymphoma, polycythemia vera, sick cell disease, IgG4 disease, HIV infection, cytomegalovirus (CMV), tuberculosis, Waldenstrom’s macroglobulinemia, multiple myeloma, cryoglobulinemia, rheumatoid arthritis, systemic lupus erythematous, and Sjogern’s syndrome.

**Table 1:** Initial laboratory work-up WBC: white blood cells; Hgb: hemoglobin; MCV: mean corpuscular volume; INR: international normalized ratio; ADP: adenosine diphosphate; SPEP: serum protein electrophoresis; dsDNA Ab: double-stranded deoxyribonucleic acid antibody; RNP: ribonucleoprotein; U3 RNP IgG: fibrillarin immunoglobulin G Reference ranges: [8]

Lab	Lab value	Reference range and unit
WBC	6.74	3.40-10.40 K/mcL
Hgb	8.7	11.5-14.9 g/dl
MCV	76.8	77.7-93.7 fl
Platelets	226	140-377 K/mcL
Sodium	142	135-145 mmol/L
Potassium	3.7	3.5-5.3 mmol/L
Chloride	116	96-108 mmol/L
Blood urea nitrogen	7	7-23 mg/dl
Creatinine	0.27	0.5-1.3 mg/dl
Alanine transaminase	147	10-45 U/L
Aspartate transaminase	88	10-40 U/L
Alkaline phosphatase	40	40-120 U/L
Bilirubin total	0.3	0.2-1.2 mg/dl
Total protein	8.9	6.2-8.1 g/dl
Albumin	1.6	3.2-5.0 g/dl
Immunoglobulin A	711	66.0-433.0 mg/dL
Immunoglobulin M	1785	42-223 mg/dL
Immunoglobulin G	4665	650-1,600 mg/dL
Immunoglobulin G subclass 4	40	1-123 mg/dL
Partial thromboplastin time	46	25-37 sec
INR	1.1	0.8-1.2
Thrombin time	18.8	16-25 sec
Fibrinogen	227	152-445 mg/dl
Von Willebrand activity	>230	40.0-163.0%
Factor IX activity	61.2	65.0-150.0%
Factor VIII activity	127.1	50.0-150.0%
Platelet function collagen/epinephrine	>300	≤180 sec
Platelets function collagen/ADP	232	≤116 sec
Viscosity	>6.34	1.10-1.80 cP
Erythropoietin	14	4-27 mU/mL
Lactate dehydrogenase	645	92-240 U/L
Haptoglobin	97	26-185 mg/dL
Direct antiglobulin antibody	Negative	Negative
Albumin SPEP	3.1	3.20-5.60 g/dl
Beta SPEP	0.8	0.50-1.10 g/dl
Alpha 1 SPEP	0.3	0.10-0.40 g/dl
Alpha 2 SPEP	0.8	0.40-1.20 g/dl
Gamma SPEP	5	0.50-1.60 g/dl
Kappa quantitative free light chains	294.59	3.30-19.40 mg/L
kappa/lambda free light chain ratio	3.19	0.26-1.65
Lambda quantitative free light chains	92.36	5.71-26.30 mg/L
C-reactive protein	<2.90	0.00-10.00 mg/L
Sedimentation rate	73	2-37 mm/hr
Anti-nuclear antibody titer	>1:640	Not detected
Anti-nuclear antibody pattern	Homogeneous nuclear pattern	Not applicable
Cyclic citrullinated peptide ab	>300	Not detected U/mL
DsDNA Ab titer	1.18055556	Not detected
Rheumatoid factor	>100	≤6.00 units
Angiotensin-converting enzyme	95	16-85 U/L
Platelets function collagen/ADP	232	≤116 sec
RNP/smith auto-antibodies	4.9	Not detected AI
U3 RNP IgG antibody	Low positive	Negative
Creatinine kinase	992	24-223 U/L

Additional hematologic labs demonstrated elevated lactate dehydrogenase, elevated von Willebrand activity, elevated platelet function collagen/epinephrine, and elevated platelet function collagen/adenosine diphosphate (ADP), all of which can be seen in platelet dysfunction. A bone marrow biopsy showed normal trilineage hematopoiesis. A fine needle aspiration biopsy of the right axillary lymph node was negative for malignant cells. An immunologic workup revealed an elevated kappa/lambda ratio, elevated immunoglobulin G (IgG), immunoglobulin M (IgM), and immunoglobulin A (IgA), and normal immunofixation. An infectious workup included a negative hepatitis panel, Epstein-Barr virus (PCR), CMV (PCR), and HIV (PCR).

Given negative infectious serologic testing, blood cultures, a normal bone marrow biopsy, a lymph node without signs of malignancy, polyclonal gammopathy consisting of IgA, IgM, and IgG, a bilateral symmetric rash, and lymphadenopathy, there was increased concern for a rheumatologic etiology.

**Figure 2:**
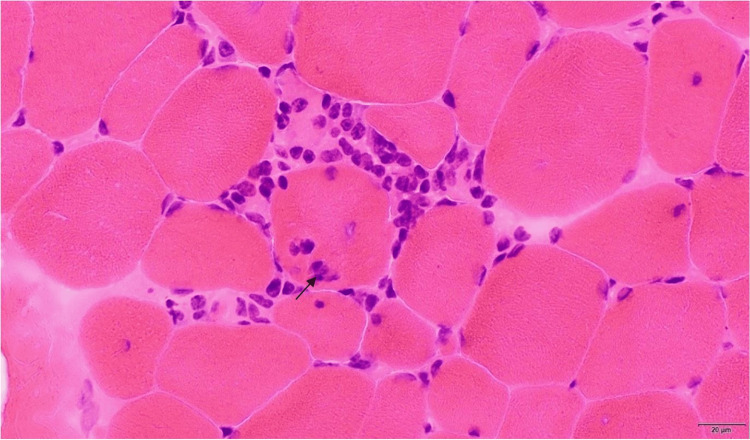
Inflammatory cells invade a viable myocyte (H&E, fresh frozen muscle, 400x magnification) Arrow shows invasion by inflammatory cells of myocytes H&E: hematoxylin and eosin

To evaluate her symptoms of muscle weakness and myopathy, a general bilateral MRI of the femurs was obtained and revealed mild increased T2 signal intensity in the bilateral semimembranosus muscles, consistent with inflammatory myositis. A muscle biopsy of the left thigh showed severe necrotizing inflammatory myopathy manifesting as lymphocytic infiltrates in the perivascular spaces of the perimysial vessels and type-specific atrophy of Type 2 myofibers, not specific to a single etiology. The differential diagnoses of the muscle biopsy pathology included overlap syndrome, antisynthetase syndrome, myositis spectrum, or dermatomyositis (Figure 2).

An autoimmune workup was then initiated and was remarkable for elevated erythrocyte sedimentation rate, positive antinuclear antibody (ANA) titer, positive Smith/ribonucleoprotein (RNP) antibodies, elevated rheumatoid factor (RF), and elevated cyclic citrullinated peptide (CCP) antibody. The extended myositis panel was positive for the RNP IgG antibody, specifically fibrillarin (U3 RNP). Several other rheumatologic antibodies were negative, as illustrated in Table 1.

Based on these results, the working diagnosis of an overlap syndrome concerning undifferentiated connective tissue disease (UCTD) and myopathy was made. Once the diagnosis of UCTD and myopathy were suspected, rheumatology recommended a 40 mg prednisone taper over 11 months. Reassuringly, her creatine kinase level and serum viscosity normalized following these interventions. The patient has not had a recurrence of her hyperviscosity symptoms over the past one and a half years. She initially had difficulty gaining weight due to myopathy and loss of muscle mass; however, since discharge, she has started regaining weight and muscle strength.

## Discussion

Hyperviscosity is a rare clinical feature of UCTD. In a review of this case report, only one other case report was found in which the patient developed hyperviscosity syndrome in UCTD. However, it was not the presenting symptom [2]. In addition, there have been studies identifying the incidence of hyperviscosity syndrome across all possible causes. Hyperviscosity is most well-studied in WM, which can be seen in up to 30% of cases [4]. There are a few reports of patients with rheumatoid arthritis or UCTD with hyperviscosity, as described in Sharif et al. [2]; however, the rheumatologic disorder had already been diagnosed prior to developing hyperviscosity [3].

Given the rarity of hyperviscosity and the overlapping nature of autoimmune diseases, pinpointing a single etiology can be challenging. This patient had positive RF, anti-CCP, double-stranded DNA, RNP/anti-Smith antibodies, and an elevated ANA titer, which suggests a picture of non-specified myositis, also known clinically as overlap syndrome. The biopsy revealed extensive complement deposition seen on endomysial capillaries and myofiber necrosis and atrophy, characteristic of dermatomyositis and anti-synthetase syndrome myositis spectra, respectively [2-3,5-7].

Hyperviscosity syndrome most commonly presents in the setting of hematologic diseases. In this case, initial labs were significant for polyclonal gammopathy, which suggests increased B-cell activation and can be seen in autoimmune disease [2-4]. Furthermore, the bone marrow biopsy ruled out hematologic etiology by showing normal trilineage hematopoiesis. Despite the elevated platelet function of collagen/epinephrine and collagen/ADP, which could indicate platelet disorders, these labs can be affected by platelet count and low hematocrit levels.

**Figure 3:**
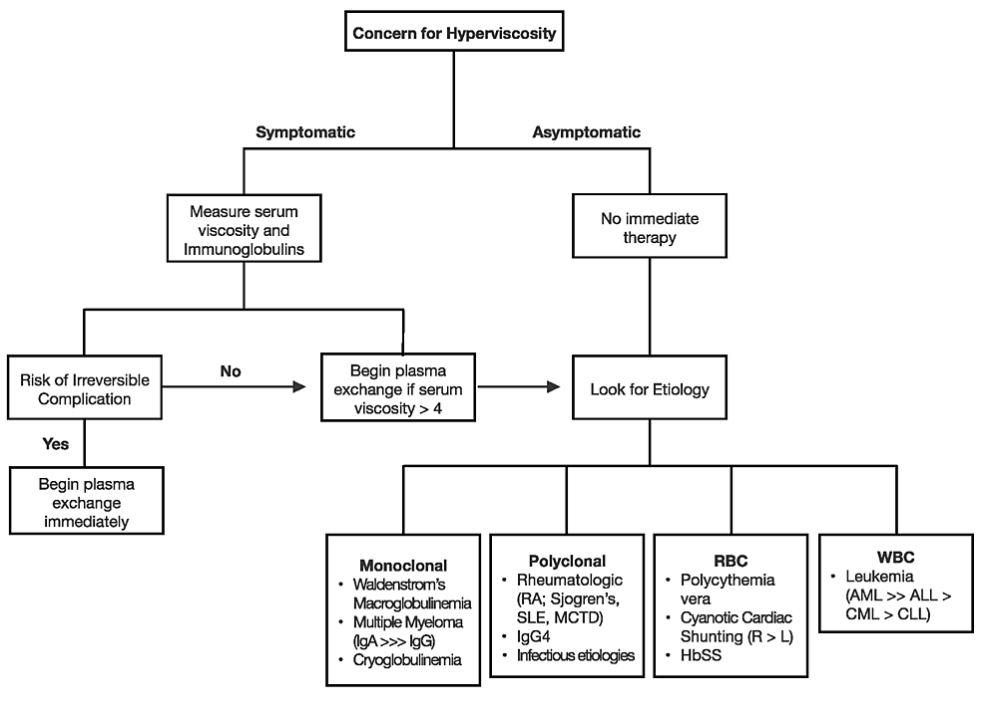
Overview of hyperviscosity evaluation and therapy Hbss: sickle cell anemia; IgG: immunoglobulin G; IgA: immunoglobulin A; RA: rheumatoid arthritis; SLE: systemic lupus erythematosus; MCTD: mixed connective tissue disease; AML: acute myelogenous leukemia; ALL: acute lymphocytic leukemia; CML: chronic myelogenous leukemia; CLL: chronic lymphocytic leukemia Image Credit: Ian Mines and Supreet Kaur (Authors)

Treatment for hyperviscosity is based on both the serum viscosity level and the symptoms caused by hyperviscosity. The overall decision-making process is outlined in Figure 3. Initial evaluation of patients with suspected or presumed hyperviscosity should consist of examining for highly specific signs of hyperviscosity or measuring serum viscosity [1]. If symptoms or signs are highly specific for hyperviscosity syndrome, therapy should begin after blood work for serum viscosity has been drawn, but without waiting for the results to be finalized. These specific and sometimes irreversible symptoms include the triad of mucosal bleeding (bilateral epistaxis, gingival bleeding, and gastrointestinal bleeding), visual disturbances (retinal hemorrhage, papilledema, and blurry vision), and neurological abnormalities (somnolence, coma, cerebral hemorrhage, seizure, and ataxia) [1]. Regardless of the etiology, either plasma, white, or red cell exchange can be used to lower blood viscosity. If the etiology is suspected to be due to a polyclonal or monoclonal etiology, plasmapheresis is the preferred therapy; however, if red blood cell or white blood cell counts are suspected of causing hyperviscosity, white or red cell exchange is required. Plasma exchange is also indicated if the patient is symptomatic with a viscosity >4 centipoises (cP). The goal of therapy is to reduce viscosity to <4 cP. If a patient is asymptomatic with elevated viscosity, targeted therapy based on the etiology is the preferred method for reducing serum viscosity [1]. This decision-making process is outlined below in Figure 3.

In this patient's case, the initial concern was hyperviscosity from the lab, which was unable to run the patient’s blood sample due to elevated viscosity. Following this, immunoglobulin and hyperviscosity levels were obtained and elevated. The patient’s ophthalmologic exam showed hemorrhages and venous dilation with venous tortuosity, which was suspected to be related to hyperviscosity. Because of both the lab findings and the ophthalmologic findings, plasma exchange was initiated. Of note, the level of hyperviscosity alone warrants plasma exchange therapy.

Apheresis (red blood cells, plasma, or leuko) is the preferred therapy to acutely lower hyperviscosity, as it directly removes components of the blood to reduce viscosity [9-11]. In this case, plasmapheresis was used to remove the patient’s antibodies to reduce her viscosity. In general, the apheresis process occurs by centrifugation of the patient’s whole blood, which leads to separation into plasma, platelet-rich plasma, white cells, and red cells. Once separated, the pathologic component of the whole blood is discarded, and the other parts are returned to the patient [9,11]. In plasmapheresis, an albumin infusion is used to replace the plasma volume that has been discarded [10-11]. The goal of plasmapheresis is to remove about 60-70% of the plasma [11]. Target amounts are also determined in leukapheresis and erythropheresis [10-11].

While apheresis is ideal for acutely lowering serum viscosity, it does not treat the etiology of the hyperviscosity, so further disease-targeted therapy is required in conjunction with apheresis to prevent further hyperviscosity.

The guidelines for the diagnosis and therapy of UCTD are not well established. Luckily, UCTD tends to respond to steroid taper and/or hydroxychloroquine [12]. Unfortunately, there are no current guidelines to determine the ideal suppressive therapy for patients with myositis in the setting of a rheumatologic disease [13]. In recent years, the use of alternative therapies alone or in conjunction with prednisone has been studied in the setting of myositis [13-14]. These include targeted immunosuppressives and immunologics, notably methotrexate, azathioprine, mycophenolate mofetil, tacrolimus, and rituximab. When the underlying cause of the myositis is known, a drug targeting the pathologic step in the inflammatory pathway is chosen [13]. While there are some promising results, due to the rarity of myositis in rheumatologic disease, the studies exploring the use of these agents are too small to draw definitive conclusions from [12-14]. Applying current literature to this case is difficult due to the diagnostic gray area in which UCTD lies [14]. It may have been reasonable in this case to trial hydroxychloroquine or rituximab in conjunction with prednisone to reduce the total dose of prednisone the patient received.

## Conclusions

A high clinical suspicion of hyperviscosity syndrome is needed for a timely and accurate diagnosis. Classically, it presents with a triad of mucosal bleeding, vision changes, and neurologic symptoms. It is critical to treat hyperviscosity in an acute setting to prevent significant complications as well as to determine the underlying disease process propagating hyperviscosity. This case demonstrated several challenges in establishing a rheumatologic etiology in patients with an uncommon presentation and a lack of classic accompanying symptoms. Rheumatologic causes present a unique challenge, as they require multiple positive findings, other etiologies must be excluded, and laboratory evaluations take weeks to complete.

While steroids remain a cornerstone treatment for rheumatologic diseases, they carry several adverse effects. Alternative immunologic or more targeted immunosuppressive therapies would be optimal; however, there are limited studies or reports on the effectiveness of newer biological therapies. Given the limited data on the use of newer immunosuppressive therapies in managing hyperviscosity syndrome, more research is needed to determine their viability to avoid the side effects of long-term steroid therapy.
